# Supplementary material for: Incidence of sinus thrombosis with thrombocytopenia—A nation-wide register study
Source: PLoS One. 2023 Feb 24;18(2):e0282226. doi: 10.1371/journal.pone.0282226 (PMC9956025; doi:10.1371/journal.pone.0282226)
Supplement: S1 Table — (DOCX) [file pone.0282226.s001.docx]

### S1 Table. Thrombosis with thrombocytopenia cases reported following ChAdOx1 nCov-19 vaccination (AstraZeneca).

| **Country** | CVST / other cases (fatal) | Age, years | Men (fatal) | Women (fatal) | Vaccinations (million) | CVST cases per million |
| --- | --- | --- | --- | --- | --- | --- |
| **Finland** | 2 (1) | < 65 | 1 (1) | 1 (0) | 0.20 | 10 |
| Norway[1] | 4/1 (3)^a^ | 32-54 (Mean 41) | 1 (0) | 4 (3) | 0.13 | 38 |
| Germany/Austria[2] | 9 / 2 (6) ^b^ | 22-49 (Md 36) | 2 | 9 | 3.0 ^c^ | 4 ^c^ |
| UK[3,4] | 102 (32) /117 (17) ^d^ | 18-79 (Median 48) | 98 | 119 | 24 | 9 |
| Germany[5] | 61 / 46 (17) ^e^ | 20-79 | 17(6) | 43(11) | 9.2 | 6.6 |
| European Economic Area[6] [7] and UK | 62 / 24 (18) ^f^  256 / … (31) | < 70 highest in 18 -24 |  | 70% women | 22 | 1.4 |

Md, median; CVST, cerebral venous sinus thrombosis
^a^ 4 CVST, 1 with intrcranial and other locations

^b^ 9 CVST, 2 other thrombosis. 5 deaths within CVST cases and 1 death within others.

^c^ Number estimated through vaccine tracker.[8]

^d^ 102 CVST and 117 other thrombosis. 32 deaths within CVST cases and 17 deaths within others Age and sex were reported for the whole cohort of 23.
^e^ 61 CVST and 46 other thrombosis. 17 deaths within CVST cases and 4 deaths within others.
^f^ 62 CVST and 24 splanchnic vein thrombosis. Number of deaths was 18 in total.

**References**

1. Schultz NH, Sørvoll IH, Michelsen AE, Munthe LA, Lund-Johansen F, Ahlen MT, et al. Thrombosis and Thrombocytopenia after ChAdOx1 nCoV-19 Vaccination. N Engl J Med. 2021 Jun 3;384(22):2124–30.

2. Greinacher A, Thiele T, Warkentin TE, Weisser K, Kyrle PA, Eichinger S. Thrombotic Thrombocytopenia after ChAdOx1 nCov-19 Vaccination. N Engl J Med. 2021 Jun 3;384(22):2092–101.

3. Scully M, Singh D, Lown R, Poles A, Solomon T, Levi M, et al. Pathologic Antibodies to Platelet Factor 4 after ChAdOx1 nCoV-19 Vaccination. N Engl J Med. 2021 Jun 10;384(23):2202–11.

4. Pavord S, Scully M, Hunt BJ, Lester W, Bagot C, Craven B, et al. Clinical Features of Vaccine-Induced Immune Thrombocytopenia and Thrombosis. N Engl J Med [Internet]. 2021 Oct 28 [cited 2022 Mar 12];385(18):1680–9. Available from: https://doi.org/10.1056/NEJMoa2109908

5. Report on suspected cases of side effects and vaccination complications after vaccination to protect against COVID-19 (reporting period December 27 to May 31, 2021) [Internet]. Paul-Erlich-Insitut; 2021 Jun. Available from: https://www.pei.de/SharedDocs/Downloads/DE/newsroom/dossiers/sicherheitsberichte/sicherheitsbericht-27-12-bis-31-05-21.html

6. EMA. European Medicines Agency. COVID-19 Vaccine AstraZeneca: benefits still outweigh the risks despite possible link to rare blood clots with low blood platelets. Mar 18 [Internet]. 2021 [cited 2021 Jul 7]. Available from: https://www.ema.europa.eu/en/news/covid-19-vaccine-astrazeneca-benefits-still-outweigh-risks-despite-possible-link-rare-blood-clots

7. Krzywicka K, van de Munckhof A, Sánchez van Kammen M, Heldner MR, Jood K, Lindgren E, et al. Age-Stratified Risk of Cerebral Venous Sinus Thrombosis After SARS-CoV-2 Vaccination. Neurology. 2022 Feb 15;98(7):e759–68.

8. European Centre for Disease Prevention and Control covid-19 VACCINE TRACKER [Internet]. [cited 2022 May 8]. Available from: https://vaccinetracker.ecdc.europa.eu/public/extensions/covid-19/vaccine-tracker.html#distribution-tab
