# Supplementary material for: Incidence of sinus thrombosis with thrombocytopenia—A nation-wide register study
Source: PLoS One. 2023 Feb 24;18(2):e0282226. doi: 10.1371/journal.pone.0282226 (PMC9956025; doi:10.1371/journal.pone.0282226)
Supplement: S2 Table — (DOCX) [file pone.0282226.s002.docx]

### S2 Table. Administered doses by Apr 2, 2021 (end of follow-up time) of each vaccine and number of COVID-19 infections by age and risk group.

| Age group | Risk group^a^ | Total | BNT162b2 | mRNA-1273 | ChAdOx1 nCov-19 | COVID-19 -infection |
| --- | --- | --- | --- | --- | --- | --- |
| 16-29 | No risk | 831712 | 16594 | 400 | 4914 | 18312 |
|  | Risk | 73269 | 2167 | 134 | 2428 | 1445 |
|  | Total | 904981 | 18761 | 534 | 7342 | 19757 |
| 30-54 | No risk | 1504207 | 59165 | 1432 | 18580 | 25178 |
|  | Risk | 246115 | 12986 | 816 | 27641 | 3812 |
|  | Total | 1750322 | 72151 | 2248 | 46221 | 28990 |
| 55-64 | No risk | 504340 | 25259 | 1000 | 13358 | 5072 |
|  | Risk | 227584 | 12680 | 1250 | 55917 | 2218 |
|  | Total | 731924 | 37939 | 2250 | 69275 | 7290 |
| 65+ | No risk | 637774 | 265211 | 24820 | 20720 | 2660 |
|  | Risk | 667912 | 320665 | 38025 | 56839 | 3363 |
|  | Total | 1305686 | 585876 | 62845 | 77559 | 6023 |
| Ages 16 and above | No risk | 3478033 | 366229 | 27652 | 57572 | 51222 |
|  | Risk | 1214880 | 348498 | 40225 | 142825 | 10838 |
|  | Total | 4692913 | 714727 | 67877 | 200397 | 62060 |

ChAdOx1 nCov-19 (Vaxzevria, AstraZeneca) BNT162b2 (Comirnaty, Pfizer–BioNTech) mRNA-1273 (Spikevax, Moderna)
^a^ A short version of a list of diseases yielding to vaccination priority. Codes were searched via registers from Jan 1, 2015 to Jan 1, 2021 and before the episode start: malignancy, type 2 Diabetes, severe lung disease, severe chronic kidney disease, History of transplantation, Down syndrome, congenital immunodeficiency, asthma, cardiovascular disease, immunosuppression*,* chronic severe liver disease, type 1 diabetes, adrenal disorder, sleep apnea, for details, see **S3 Table.** The administered dose counts in the Vaccination register were in high accordance with vaccine dose count from a different source, the dose count from centralized distribution (internal information, THL).
