# Supplementary material for: Incidence of sinus thrombosis with thrombocytopenia—A nation-wide register study
Source: PLoS One. 2023 Feb 24;18(2):e0282226. doi: 10.1371/journal.pone.0282226 (PMC9956025; doi:10.1371/journal.pone.0282226)
Supplement: S3 Table — (DOCX) [file pone.0282226.s003.docx]

### S3 Table. Codes for diseases in registers.

| Previous disease |
| --- |
| Malignancy^a^ (ICD-10: C00, C01, C20-C43, C45-C97, D05.1, D39, *SII :* *115, 116, 117, 128, 130)* |
| Cardiovascular disease^a^ including stroke, heart failure, coronary heart disease, complicated hypertension (ICD-10: I11.0, I11.9, I12, I13.0, I13.1 I13.2, I13.9, I15, I20-I25, I50, I60-I64, I69, *SII :* *201, 206, 205)* |
| Severe lung disease^a^ ICD-10: any J41- J47, *SII:* *203* |
| Type 2 diabetes (ICD-10: E11, E13, E14, ICPC-2: T90, medicine purchase register, ATC-code: A10B) |
| Risk group for COVID-19 vaccination in the Finnish vaccination campaign consists of diseases with highest COVID-19 morbidity and mortality[1,2] and served as indication for vaccination with ChAdOx1 nCov-19 during the study period. Included in the list during analysis of this study were, from above, Malignancy, Cardiovascular disease (excluding Chronic hypertension I11.9 *SII :* *205*, including Pericarditis I30, I32), Severe lung disease (except *SII :* *203,* including ICPC-2 code R96), Type 2 Diabetes. In addition, the following Diabetes with nephropathy (ICD-10: E10.2, E11.2, E14.2), Severe chronic kidney disease (ICD-10: N00-N08, N11, N14, N18, N19), History of transplantation (ICD-10: T86, Z94, *SII :* *127*), Down syndrome (ICD-10: Q90), Congenital immunodeficiency (ICD-10: D70.81, D70.89, D80-D84, E31.00), Sequelae of head trauma (ICD-10: T90), Chronic severe liver disease (ICD-10: K70.2-K70.4, K71-K74), Other diabetes (ICD-10: E10, E14, ICPC-2 code T89 or from medicine purchase register ATC-code A10A), Adrenal disorders (ICD-10: E25.0, E27.1,E27.2, E27.4, E31.0, E89.6, P72, *SII :* *105*), Sleep apnea (ICD-10: G47.3, procedure codes WX723 and WX780), Clozapine therapy (ATC: N05AH02), Psycothic disorders (ICD-10: F20-F29), Paralyses or muscle disorders (ICD-10: G70, G71 - G73, G80-G83). |
| No risk: Not in the Risk group |
| Disease during episode |
| Cerebral infarction (ICD-10: I63.6, G45, G46, I63, I64) |
| Cranial surgery (operation codes AAA – AAW)  Cranial trauma (ICD-10: S02-S07)  Cranial tumor (ICD-10: C70-C71, C79.3, D32.0, D33, D35.2, D35.30) |

Abbreviations: International Classification for Diseases, 10^th^ edition (ICD-10), International Classification for Primary Care, 2^nd^ edition (ICPC-2 codes), Nordic Classification of Surgical Procedures (NCSP) or Social Insurance Institute disease codes (*SII,* *italics*). All codes from register notifications dated after Jan 1, 2015 to Jan 1^st^ 2021 or the first event of cerebral venous sinus thrombosis. Data came from the Care registers for health care and from the Reimbursements for medical expenses -register.

^a^ ICD-10 data came from specialized care only.

**References**

1. Salo H, Lehtonen T, Auranen K, Baum U, Leino T. Predictors of hospitalisation and death due to SARS-CoV-2 infection in Finland: a population-based register study with implications to vaccinations [Internet]. medRxiv; 2021 Jul [cited 2022 Mar 4] p. 2021.07.04.21259954. Available from: https://www.medrxiv.org/content/10.1101/2021.07.04.21259954v1

2. Poukka E, Baum U, Palmu AA, Lehtonen TO, Salo H, Nohynek H, et al. Cohort study of Covid-19 vaccine effectiveness among healthcare workers in Finland, December 2020 - October 2021. Vaccine [Internet]. 2022 Jan 31 [cited 2022 Feb 8];40(5):701–5. Available from: https://www.ncbi.nlm.nih.gov/pmc/articles/PMC8683266/
