# Supplementary material for: Incidence of sinus thrombosis with thrombocytopenia—A nation-wide register study
Source: PLoS One. 2023 Feb 24;18(2):e0282226. doi: 10.1371/journal.pone.0282226 (PMC9956025; doi:10.1371/journal.pone.0282226)
Supplement: S4 Table — (DOCX) [file pone.0282226.s004.docx]

### S4 Table. Case Report.

| Case number | 1 | 2 |
| --- | --- | --- |
| Age (years) | 21 | 39 |
| Gender (M/F) | F | M |
| CVST | Yes | Yes |
| ICH | No | Yes |
| Other thromboses | No | AMI |
| Symptoms onset (days) | 9 | 7 |
| Heparin/LMWH treatment | No | No |
| Platelet count nadir (per mm^3^) | 50,000 | 20,000 |
| D-dimer peak (mg/liter) | 14 | > 128 |
| Platelet factor 4 antibodies^a^ | Yes | Yes |
| Previous PCR-positive COVID-19 infection | No | No |
| Outcome | Recovered | Fatal on day 5 |

CVST, Cerebral venous sinus thrombosis; LMWH, Low-molecular-weight heparin prior to CSVT and thrombocytopenia.

AMI, Myocardial infarction.

ICH, Intracerebral hemorrhage.

^a^Antibodies to platelet factor 4 (PF4) were tested both with gel

agglutination based rapid assay (ID-PaGIA Heparin/PF4 Antibody Test,

Bio-Rad Laboratories, United States) and enzyme-linked immunosorbent

assay (ELISA) (Asserachrom HPIA, Diagnostica Stago, France). The rapid

assay was negative while the ELISA test was positive in both

cases.

### Observed cases

**By Apr 2, 2021, 200,000 persons had received ChAdOx1 nCov-19 vaccination. The two confirmed CVST with thrombocytopenia cases were detected from the registers as described and, additionally, from the reports to the Finnish Medicines Agency, FIMEA. The descriptions and case reports of these individuals are described in detail elsewhere. In brief, a woman aged 21 years and a man in aged 39. Both had several concomitant diseases, male deceased on day 5 and the female recovered.**
