# Supplementary material for: Incidence of sinus thrombosis with thrombocytopenia—A nation-wide register study
Source: PLoS One. 2023 Feb 24;18(2):e0282226. doi: 10.1371/journal.pone.0282226 (PMC9956025; doi:10.1371/journal.pone.0282226)
Supplement: S5 Table — (DOCX) [file pone.0282226.s005.docx]

### S5 Table. Total population by exposure, age group and sex.

|  | **Population** | | | **BNT162b2** | | | **mRNA-1273** | | | **ChAdOx1 nCov-19** | | | **COVID-19 -infection** | | |
| --- | --- | --- | --- | --- | --- | --- | --- | --- | --- | --- | --- | --- | --- | --- | --- |
| Age | Male | Female | Total | Male | Female | Total | Male | Female | Total | Male | Female | Total | Male | Female | Total |
| 0-15 | 448632 | 429070 | 877702 | 117 | 106 | 223 | 25 | 17 | 42 | 27 | 31 | 58 | 5948 | 5403 | 11351 |
| 16-29 | 466016 | 438965 | 904981 | 4345 | 14416 | 18761 | 177 | 357 | 534 | 2498 | 4844 | 7342 | 10329 | 9428 | 19757 |
| 30-54 | 899083 | 851238 | 1750321 | 15999 | 56152 | 72151 | 756 | 1492 | 2248 | 20091 | 26130 | 46221 | 15092 | 13898 | 28990 |
| 55-64 | 363239 | 368685 | 731924 | 7902 | 30037 | 37939 | 992 | 1258 | 2250 | 36323 | 32952 | 69275 | 3696 | 3594 | 7290 |
| 65+ | 578059 | 727627 | 1305686 | 239840 | 346036 | 585876 | 25701 | 37144 | 62845 | 40058 | 37501 | 77559 | 2707 | 3316 | 6023 |
| Subtotal^a^ | 2306397 | 2386515 | 4692912 | 268086 | 446641 | 714727 | 27626 | 40251 | 67877 | 98970 | 101427 | 200397 | 31824 | 30236 | 62060 |
| Total | 2755029 | 2815585 | 5570614 | 268203 | 446747 | 714950 | 27651 | 40268 | 67919 | 98997 | 101458 | 200455 | 37772 | 35639 | 73411 |

ChAdOx1 nCov-19 (Vaxzevria, AstraZeneca) BNT162b2 (Comirnaty, Pfizer–BioNTech), mRNA-1273 (Moderna).

^a^Subtotal includes ages 16 and above.
