# Supplementary material for: Incidence of sinus thrombosis with thrombocytopenia—A nation-wide register study
Source: PLoS One. 2023 Feb 24;18(2):e0282226. doi: 10.1371/journal.pone.0282226 (PMC9956025; doi:10.1371/journal.pone.0282226)
Supplement: S6 Table — Estimates and 95% confidence intervals. (DOCX) [file pone.0282226.s006.docx]

### S6 Table. Incidences per 100,000 person years of cerebral venous sinus thrombosis and thrombocytopenia during unexposed time. Estimates and 95% confidence intervals.

|  | **Males** | **Females** | **Both sexes** |
| --- | --- | --- | --- |
| CVST, register based^a^ |  |  |  |
| 0-15 | 0.36 (0.04– 1.28) | 0.93 (0.30– 2.17) | 0.64 (0.26– 1.31) |
| 16-29 | 1.03 (0.38– 2.24) | 2.19 (1.13– 3.83) | 1.59 (0.94– 2.51) |
| 30-54 | 1.69 (1.02– 2.64) | 2.84 (1.92– 4.06) | 2.25 (1.66– 2.98) |
| 55-64 | 3.56 (2.03– 5.78) | 3.53 (2.02– 5.73) | 3.54 (2.42– 5.00) |
| 65+ | 2.77 (1.67– 4.33) | 4.07 (2.84– 5.66) | 3.49 (2.63– 4.56) |
| All ages | 1.82 (1.40– 2.33) | 2.84 (2.30– 3.46) | 2.33 (1.99– 2.72) |
| CVST, confirmed^b^ |  |  |  |
| 0-15 | 0.18 (0.00– 0.99) | 0.56 (0.11– 1.63) | 0.36 (0.10– 0.93) |
| 16-29 | 0.86 (0.28– 2.00) | 1.83 (0.88– 3.36) | 1.33 (0.74– 2.19) |
| 30-54 | 1.43 (0.81– 2.31) | 1.80 (1.08– 2.81) | 1.61 (1.12– 2.24) |
| 55-64 | 2.67 (1.38– 4.66) | 2.21 (1.06– 4.06) | 2.44 (1.53– 3.69) |
| 65+ | 2.04 (1.12– 3.43) | 2.79 (1.79– 4.15) | 2.46 (1.74– 3.38) |
| All ages | 1.41 (1.04– 1.87) | 1.91 (1.48– 2.43) | 1.66 (1.37– 2.00) |
| CVST, confirmed, with thrombocytopenia^c^ |  |  |  |
| 0-15 | 0.18 (0.00– 0.99) | 0.37 (0.04– 1.34) | 0.27 (0.06– 0.80) |
| 16-29 | 0.17 (0.00– 0.95) | 0.00 (0.00– 0.67) | 0.09 (0.00– 0.49) |
| 30-54 | 0.09 (0.00– 0.50) | 0.00 (0.00– 0.35) | 0.05 (0.00– 0.26) |
| 55-64 | 0.89 (0.24– 2.28) | 0.44 (0.05– 1.59) | 0.66 (0.24– 1.45) |
| 65+ | 0.58 (0.16– 1.49) | 0.12 (0.00– 0.65) | 0.32 (0.11– 0.76) |
| All ages | 0.32 (0.16– 0.58) | 0.14 (0.05– 0.34) | 0.23 (0.13– 0.38) |

ChAdOx1 nCov-19 (Vaxzevria, AstraZeneca), **BNT162b2 (Comirnaty, Pfizer–BioNTech),** NA not applicable, for those under 16 years the COVID‑19 vaccines were unavailable.

^a^ CVST, cerebral venous sinus thrombosis: As a main diagnosis either of ICD-10 codes I636, I676, or G08 included. Only emergency-room visits and non-scheduled in-patient hospitalizations were included.

^b^ CVST, confirmed: Episodes in registers that were confirmed by chart review (clinical radiological reports and clinical interpretations).

^c^ CVST, confirmed, with thrombocytopenia: Platelet count < 150,000 per cubic millimeter within 14 days before and after episode start.

Note that the 28-day risk time after **BNT162b2 was free from** episodes with confirmed CVST. **Note also, that risk time after mRNA-1273 (Moderna) was free from any CVST.**

**For incidence per million per 28 days, see Table 1 in main article.**
