# Supplementary material for: Incidence of sinus thrombosis with thrombocytopenia—A nation-wide register study
Source: PLoS One. 2023 Feb 24;18(2):e0282226. doi: 10.1371/journal.pone.0282226 (PMC9956025; doi:10.1371/journal.pone.0282226)
Supplement: S7 Table — (DOCX) [file pone.0282226.s007.docx]

### S8 Table. Possible etiology of the confirmed cerebral venous sinus thrombosis with thrombocytopenia cases.

| Etiological group | Count | Examples for reasons |
| --- | --- | --- |
| TP at time of CVST and no obvious other reason | 9 | No obvious other reason |
| TP due to previous disease | 3 | Mastoiditis, cranial operation and/or trauma |
| TP due to other known reason | 0 |  |
| Very short duration of TP | 2 | Suspected heparin associated thrombocytopenia |
| Not enough information to suggest any TP etiology | 4 | TP during delirium, very quick correction of TP, adenoviral infection, or tumor as etiology? |

TP = thrombocytopenia
