# Supplementary material for: Incidence of sinus thrombosis with thrombocytopenia—A nation-wide register study
Source: PLoS One. 2023 Feb 24;18(2):e0282226. doi: 10.1371/journal.pone.0282226 (PMC9956025; doi:10.1371/journal.pone.0282226)
Supplement: S1 Text — A detailed description of Bayesian estimation methodology. (DOCX) [file pone.0282226.s008.docx]

# Incidence of Sinus Thrombosis with Thrombocytopenia - a Nation-wide Register Study

Petteri Hovi, MD, PhD, Arto A. Palmu, MD, PhD, Tuomo Nieminen, MSc,

Miia Artama, MSc, PhD, Jukka Jokinen, PhD, Esa Ruokokoski, MSc, Riitta Lassila MD, PhD, Hanna Nohynek MD, PhD, Terhi Kilpi, MD, PhD

## Supplemental methods

This appendix has been provided by the authors to give readers additional information about their work.

Supplement to: Hovi P et al.

### Statistical methods

We used Bayesian methods to estimate parameters of the Poisson regression model described in the statistical methods section. Inference was based on values sampled from the joint posterior distribution of the parameters. Credible intervals were derived from the quantiles of the posterior samples after appropriate transformations (such as the exponent function). A more complicated transformation was used to derive posterior samples for the exposure attributable risk, which was treated as a truncated distribution with non-negative values. For each age and gender group with data in the exposed state, and for each sample drawn from the joint posterior distribution of the parameters, we predicted the outcome incidence from the regression equation. We summed the predicted incidences over the age and gender groups to get a posterior sample for the incidence in the exposed group. We repeated this over all samples from the joint posterior distribution. We then changed the exposure indicator to 0 and did the same. Then for each pair of samples between the different exposure states, we computed the difference in incidence via subtraction which resulted in posterior samples for the exposure attributable risk. We assigned values < 0 to zero, assuming that the incidence is at least as high for the exposed than the not exposed.

We used a N(0,10) prior distribution for all regression coefficients. The event in question is rare in the general population and so for estimation efficiency we used a vaguely informative N(-1,3) prior for the intercept. We treat time as 100K person year units and our intercept prior distribution choice corresponds to the prior expectation of exp(-1) = 0.37 events per 100K person years for the reference sex and age group during the unexposed state. The reference sex was male and the reference age group was the youngest.

We carried out sensitivity analyses using different standard deviation choices for the regression coefficient prior distributions and also compared the chosen vaguely informative intercept prior with a more uninformative N(0, 10) prior. In the sensitivity analysis we monitored the impact of the prior distribution choices to the main quantities of interest: the posterior quantiles of the regression coefficient for the exposure state indicator. We also compared the posterior quantiles to a frequentist maximum likelihood estimate.

Sensivity analysis results: Regression coefficient prior distribution choices with smaller standard deviations provided lower posterior quantiles as they truncated the distribution more heavily towards 0. Prior distributions with standard deviations 10, 5, 3 and 2 (and mean 0) produced posterior medians 41, 37, 31 and 22 in the exponent scale, respectively. The frequentist maximum likelihood solution was 47 and the chosen N(0, 10) prior distribution provided similar but slightly lower estimate to the frequentist solution. The choice of the intercept prior distribution had no impact to the main quantity of interest. The estimation was carried out using STAN (Stan Development Team. 2019. Stan Modeling Language Users Guide and Reference Manual, 2.21. <https://mc-stan.org>) and R (R Core Team (2020). R: A language and environment for statistical computing. R Foundation for Statistical Computing, Vienna, Austria. URL https://www.R-project.org/).
