# Supplementary material for: Incidence of sinus thrombosis with thrombocytopenia—A nation-wide register study
Source: PLoS One. 2023 Feb 24;18(2):e0282226. doi: 10.1371/journal.pone.0282226 (PMC9956025; doi:10.1371/journal.pone.0282226)
Supplement: Attachment — Submitted filename: Response to the Reviewers 2022-08-20b.docx [file pone.0282226.s009.docx]

# Response to Reviewers

PONE-D-22-09809

Incidence of Sinus Thrombosis with Thrombocytopenia - a Nation-wide Register Study

PLOS ONE

Dear Editor,

We thank your team including the Reviewers for their work on our manuscript and are happy to submit a revised manuscript. As requested, we respond to each of your comments, and we now include both 'Revised Manuscript with Track Changes and an unmarked version of our revised paper ‘Manuscript’. We include the Editors’ and Reviewers’ comments in *italics* within quotes.

*“Journal Requirements:*

*When submitting your revision, we need you to address these additional requirements.*

*1. Please ensure that your manuscript meets PLOS ONE's style requirements, including those for file naming. The PLOS ONE style templates can be found at* 
[*https://journals.plos.org/plosone/s/file?id=wjVg/PLOSOne_formatting_sample_main_body.pdf*](https://journals.plos.org/plosone/s/file?id=wjVg/PLOSOne_formatting_sample_main_body.pdf) *and* 
[*https://journals.plos.org/plosone/s/file?id=ba62/PLOSOne_formatting_sample_title_authors_affiliations.pdf*](https://journals.plos.org/plosone/s/file?id=ba62/PLOSOne_formatting_sample_title_authors_affiliations.pdf)*”*

Response 1: We have revised the manuscript and files. This includes revision of the filenames for figures and supplemental files and added a caption to our supplemental methods file to the “Supporting information”

*2. “Thank you for stating the following in the Acknowledgments Section of your manuscript:*

*The current study received no external funding outside THL and there are no conflicting interests. Finnish Institute for Health and Welfare (THL) conducts Public-Private Partnership with vaccine manufacturers and has received research funding from Sanofi Inc., Pfizer Inc., and GlaxoSmithKline Biologicals SA.  AAP, TN, JJ, ER, and TK have been investigators in these studies, but they have received no personal remuneration. P.H. reports being, in 2016 and 2017, a member in an expert group “Future Rheumatology Advisory Board”, funded by Pfizer.*

*We note that you have provided funding information that is not currently declared in your Funding Statement. However, funding information should not appear in the Acknowledgments section or other areas of your manuscript. We will only publish funding information present in the Funding Statement section of the online submission form. 
Please remove any funding-related text from the manuscript and let us know how you would like to update your Funding Statement. Currently, your Funding Statement reads as follows:*

*The authors received no specific funding for this work*

*Please include your amended statements within your cover letter; we will change the online submission form on your behalf.”*

Response 2: We have removed our funding information from “acknowledgements”. Our amended funding statement: “The current study received no external funding outside THL and there are no conflicting interests. Finnish Institute for Health and Welfare (THL) conducts Public-Private Partnership with vaccine manufacturers and has received research funding from Sanofi Inc., Pfizer Inc., and GlaxoSmithKline Biologicals SA.  A.A.P., T.N., J.J., E.R., and T.K. have been investigators in these studies, but they have received no personal remuneration. P.H. reports being, in 2016 and 2017, a member in an expert group “Future Rheumatology Advisory Board”, funded by Pfizer Inc.”

3. *“Thank you for stating the following in the Competing Interests section:*

*The current study received no external funding outside THL and there are no conflicting interests. Finnish Institute for Health and Welfare (THL) conducts Public-Private Partnership with vaccine manufacturers and has received research funding from Sanofi Inc., Pfizer Inc., and GlaxoSmithKline Biologicals SA.  AAP, TN, JJ, ER, and TK have been investigators in these studies, but they have received no personal remuneration. P.H. reports being, in 2016 and 2017, a member in an expert group “Future Rheumatology Advisory Board”, funded by Pfizer.*

*Please confirm that this does not alter your adherence to all PLOS ONE policies on sharing data and materials, by including the following statement: "This does not alter our adherence to  PLOS ONE policies on sharing data and materials.” (as detailed online in our guide for authors* [*http://journals.plos.org/plosone/s/competing-interests*](http://journals.plos.org/plosone/s/competing-interests)*).  If there are restrictions on sharing of data and/or materials, please state these. Please note that we cannot proceed with consideration of your article until this information has been declared.*

*Please include your updated Competing Interests statement in your cover letter; we will change the online submission form on your behalf.”*

Response 3: Our updated Competing interest statement. “The current study received no external funding outside THL and there are no conflicting interests. Finnish Institute for Health and Welfare (THL) conducts Public-Private Partnership with vaccine manufacturers and has received research funding from Sanofi Inc., Pfizer Inc., and GlaxoSmithKline Biologicals SA.  A.A.P., T.N., J.J., E.R., and T.K. have been investigators in these studies, but they have received no personal remuneration. P.H. reports being, in 2016 and 2017, a member in an expert group “Future Rheumatology Advisory Board”, funded by Pfizer Inc. This does not alter our adherence to PLOS ONE policies on sharing data and materials. However, by Finnish law, the authors are not permitted to share individual-level register data.”

*4. “In your Data Availability statement, you have not specified where the minimal data set underlying the results described in your manuscript can be found. PLOS defines a study's minimal data set as the underlying data used to reach the conclusions drawn in the manuscript and any additional data required to replicate the reported study findings in their entirety. All PLOS journals require that the minimal data set be made fully available. For more information about our data policy, please see* [*http://journals.plos.org/plosone/s/data-availability*](http://journals.plos.org/plosone/s/data-availability)*.*

*"Upon re-submitting your revised manuscript, please upload your study’s minimal underlying data set as either Supporting Information files or to a stable, public repository and include the relevant URLs, DOIs, or accession numbers within your revised cover letter. For a list of acceptable repositories, please see* [*http://journals.plos.org/plosone/s/data-availability#loc-recommended-repositories*](http://journals.plos.org/plosone/s/data-availability#loc-recommended-repositories)*. Any potentially identifying patient information must be fully anonymized.*

*Important: If there are ethical or legal restrictions to sharing your data publicly, please explain these restrictions in detail. Please see our guidelines for more information on what we consider unacceptable restrictions to publicly sharing data:* [*http://journals.plos.org/plosone/s/data-availability#loc-unacceptable-data-access-restrictions*](http://journals.plos.org/plosone/s/data-availability#loc-unacceptable-data-access-restrictions)*. Note that it is not acceptable for the authors to be the sole named individuals responsible for ensuring data access.*

*We will update your Data Availability statement to reflect the information you provide in your cover letter.”*

Response 4: Our updated Data Availability statement: ”A minimal dataset is available in Table 1 in the manuscript. It displays the event counts and incidences for each combination of age-group, sex, and exposure status.”

*5. PLOS requires an ORCID iD for the corresponding author in Editorial Manager on papers submitted after December 6th, 2016. Please ensure that you have an ORCID iD and that it is validated in Editorial Manager. To do this, go to ‘Update my Information’ (in the upper left-hand corner of the main menu), and click on the Fetch/Validate link next to the ORCID field. This will take you to the ORCID site and allow you to create a new iD or authenticate a pre-existing iD in Editorial Manager. Please see the following video for instructions on linking an ORCID iD to your Editorial Manager account:* [*https://www.youtube.com/watch?v=_xcclfuvtxQ*](https://www.youtube.com/watch?v=_xcclfuvtxQ)

Response 5: The corresponding author’s ORCHID number 0000-0002-7790-007X is now successfully attached to the submission.

## Reviewer #1 - 1

*“The introduction of thrombosis in unusual regions, including cerebral venous sinus thrombosis(CVST), by SARS-CoV-2 vaccines, caused a major public concern. Although vaccine-induced CVST is rare, for instance, 0.55 per 100,000 person-months in the first month after 7,126,434 vaccination in Germany, the affliction of young and previously healthy people makes it a critical area of research.*

*This is a cohort study to investigate the association between COVID-vaccination and the evolution of CVST. Authors showed that only 2 out of 117 patients with CVST fulfill the criteria of CVST associated with vaccine-induced thrombosis and thrombocytopenia(VITT). In the other word, the rate of CVST-VITT was about 1 per 100.00 adult population.* 

*Research design is sound. Methods are adequately described, and results are clearly presented. Some significant issues should be addressed, though:*

*Method:
-“ The date of the first contact to hospital resulting in a relevant CVST diagnosis indicated disease onset” seems to be obscure. The authors should clearly differentiate between the date of admission and the date of first clinical presentation ( the date of onset)
“*

Response #1 - 1. For more clarity, we made the following editions (from line 71, Methods, Note: line numbers refer to the file with tracked changes):

“We were unable to systematically collect data on symptom onset. The date of the first contact to hospital resulting in a relevant CVST diagnosis, with or without admission, indicated disease episode onset, and a new CVST episode could start at the earliest 28 days after visit / discharge. We included all relevant data provided in care notifications sent from the caregivers to the register, from the first visit to the hospital (usually emergency room) and/or admission (episode start) until discharge.”

*Reviewer #1 - 2*

*-“Original radiological images were not reviewed.”. it should be considered a drawback.*

Response #1 -2: We have now considered this, see Response #1 -7 below.

*Reviewer #1 - 3*

*“- It should be mentioned that using the nationwide registry supervised by reviewers decreased the risk of false positives; however, some false-negative cases, particularly mild cases of CVST with an isolated headache, might be overlooked.”*
Response #1 -3: We agree, and also our manuscript states (Discussion, line 280): “…cases with mild symptoms may have been missed.” We currently have increased wording explaining the relevance of this kind of false-negative findings: ” It is unlikely that these kinds of severe cases would be missed in our setting, irrespective of the vaccination status.”

*Reviewer #1 - 4*

*“-Were there any patients diagnosed only by antigen detection method rather than PCR?”*

Response #1 -4. We changed the numbers to tell about cases and not of the tests (Methods\ SARS CoV-2 infections, line 100): “

During the study period, test positivity with absence of positive tests during the preceeding 12 months indicated a distinct COVID-19 case. Real-time reverse-transcription polymerase chain reaction (PCR) (involved in 97.7% of all cases) was the most common test, while 2.3% of the cases based solely on the antigen detection method. The testing capacity exceeded 3,000 daily samples per million inhabitants since August, 2020.[16]

“

*Reviewer #1 - 5*

*“Results

-“We detected 167 register-based CVST episodes in 156 subjects”. Seven percent risk of recurrence in a 15-month period of observation is unusual. Authors should justify this result.
“*

Response #1 – 5: We agree. The high percentage is due to the fact that our definition for CVST was aimed to be inclusive: “a new CVST episode could start at the earliest 28 days after visit / discharge.” (Methods, Line 73).

That definition based on register data only and was unable to correctly detect CVST as evaluated against our chart review based ‘confirmed CVST’ -definition. We thus emphasize the importance and usefulness of this ‘confirmed CVST’ -definition. To the current manuscript, we added (Discussion, line 251): “In our data, 2 of the 117 patients presented with more than a single [confirmed CVST] episode during the study period.”

*Reviewer #1 - 6*

*“-Table-2
Concomitant diseases, which were mentioned in table-2 are not the most important predisposing factors for CVST. Provocative predisposing factors for CVST include pregnancy, puerperium, OCPs, infections, head injury, and dehydration. Non-provocative factors include inherent conditions such as acquired or genetic thrombophilia, malignancies, hematologic diseases, and inflammatory diseases. Some of them, such as SLE, antiphospholipid antibody syndrome, leukemia, and some medications, can induce both CVST and thrombocytopenia; and should be excluded for diagnosing VITT. However, they were not mentioned in the manuscript.
- The status of anti-platelet factor 4(PF4) antibodies was mentioned*
“

Response #1 – 6: Table 2 summarizes factors that could be confounders. The “Concominant diseases” in Table 2 are known to associate with a higher CVST incidence and individuals with these conditions were also give priority in the vaccination program that was employed during the study period. The Reviewer correctly lists a number of states and diseases that indeed predispose to CVST. Pregnancy, puerperium, oral contraceptive pills (OCPs), infections, head injury, dehydration, however, were not expected to increase the likelihood of being vaccinated during the study period. Please note, that head injury as well as other states and diseases brought up by the Reviewer, are included in Table 2, see row “Other risk group^b^ “, with ^b^ referring to S3 Table.

Precision: We only detected two cases and were unable to perform formal analysis that take concomitant disease into account. In Discussion, line 284, we have edited:

“We found 2 cases of CVST with thrombocytopenia after vaccination of 200,000 subjects with ChAdOx1 nCov-19 adeno-virus-vector vaccine. Since the vaccine was withdrawn from subjects younger than 65, a longer catchment period would hardly have increased the number of vaccinated cases or the limited precision of our vaccine adverse effect analysis.”

Confounding:In Discussion, line 293, we have added a sentence : “

Due to the vaccination prioritisation, most ChAdOx1 nCov-19 adeno-virus-vector vaccine doses were administered to subjects with comorbidities, which raises the question of bias in the risk estimates**.** If such comorbidities predisposed to CVST with thrombocytopenia, this would result in overestimation of vaccine-attributable risk. Yet, a considerably higher estimate has been reported from Norway in health care professionals[2].”

Clearly, excluding subjects with certain diseases that can associate with both sinusthrombosis and thrombocytopenia before diagnosis of VITT may, for many purposes, be the method of choice, as this increases the likelihood of vaccine as the etiology. In our study, however, the aim was to employ the same definition during unvaccinated time and vaccinated time and show how rare the entity in question is.

*Reviewer #1 -7*

*“Discussion:
The discussion part is too long.
Limitations of the study were not mentioned.
“*

Response #1 -7

We have constructed the Discussion by adding the changes requested by the reviewers and by reducing the text. We deleted whole sentences in Discussion, lines 242 – 246. If the Editor and / or Reviewers so request, we are happy to reduce the text more.

Regarding the limitations, we edited accordingly a paragraph in Discussion (line 276), partially referred to also in response #1 -3.:

”Our study has weaknesses. As any other vaccination surveillance study, also ours may be biased by the increase of public attention on an adverse effect. CVST typically presents with severe symptoms including intense and protracted headache. However, atypical CVST cases with mild symptoms may have been missed. Nevertheless, the CVST cases reported after adenoviral vector vaccination have had severe and clear symptoms. It is unlikely that these kinds of severe cases would be missed in our setting, irrespective of the vaccination status. Also, our definitions based on clinicians’ decisions and routine evaluation including the radiology. This might decrease our precision, but only slightly, because of Finland’s setting with equal access to our high-quality health care system.”

## Reviewer #2 -1:

*“Thrombosis with thrombocytopenia syndrome (TTS) has been reported after vaccination with the adenoviral vector based SARS-CoV-2 vaccines ChAdOx1 nCov-19 (Oxford–AstraZeneca) and Ad26.COV2.S (Janssen/Johnson & Johnson). Using nationwide registers, the authors estimated the incidence of CVT and CVT with thrombocytopenia since 2020 to established whether vaccines against SARS-CoV-2 were associated with increased risk of CVT with thrombocytopenia.
Comments and suggestions are listed below:
1. Thrombocytopenia is a key feature of the vaccine-induced immune thrombotic thromcytopenia syndrome (VITT). However, the authors have not addressed this in their analysis of ADRs. This should be acknowledged as a major limitation.
“*

Response #2 -1: We agree that thrombocytopenia is an important feature of VITT and TTS and the primary aim of the study was to evaluate the background incidence of this syndrome (CVST with thrombocytopenia). In the Introduction we mention thrombocytopenia as one of the features of TTS. As the part of our description of methods of our adverse drug reaction (ARD) analysis (line 55) we state: “The primary outcome of interest was confirmed CVST with thrombocytopenia.”

Also in our Results we mention “the two that were confirmed to be CVST also coincided with thrombocytopenia (line 213).“ In order to clarify the situation, we have now included a new sentence in Discussion, Line 232:

The term vaccine-induced immune thrombotic thrombocytopenia (VITT) has gained widespread use for TTS following vaccination since its recognition in spring 2021.[23]

*Reviewer #2 -2:*

“The authors say in the results that CVT occurring within 28 days was considered. However, this is not detailed in the methods. Case definition and adjudication should be better described in the methods section.
“

Response #2 -2: We state in the manuscript, under subtitle “Exposed and unexposed follow-up periods”, in Methods, line 59, the definition of our 28-day risk period. The register based CVST / confirmed CVST and confirmed CVST with thrombocytopenia are explained under relevant subheadings in Methods, lines 77, 82 and 93. For clarity, we added the words “Confirmed CVST”, on rows 93 and 95.

*Reviewer #2 -3:*
“the number of events is small (only 2 patients with CVT and thrombocytopenia after vaccination, only 200.000 subjects vaccinated with ChAdOx 1 nCov-19). For this reason, there is substantial imprecision in the estimates that are provided (very large confidence intervals). The discussion on the study limitations should be expanded.
“

Response #2 -3: We have expanded Discussion on the limited power affecting this portion of our results (Response #1 – 6, above)

Reviewer #2 -4*.*

*Three physicians revised all suspected clinical cases. Please explain whether these clinicians were neurologists or physicians with a special expertise in cerebrovascular diseases.*

*Response #2 -4:* In the Methods, line 85, we have made edited text: “Three clinically experienced physicians, two pediatricians and one epidemiologist with a generalist background (P.H., A.A.P. and T.K.) independently reviewed all cases. In addition, please note line 95 “One expert hematologist (R.L.) confirmed the episodes without a chronic disease as a potential etiology for thrombocytopenia.”

*Reviewer #2 -5.*

*If possible, the authors should consider describing what were the causes/comorbidities in patients with thrombocytopenia and CVT, particularly those that may justify concomitant thrombocytopenia. Information on the range of platelet values would be also useful (less severe thrombocytopenia in patients without VITT?)*

Response #2 -5. Unfortunately, the Finnish law sets limitations, and we cannot reveal full case presentations of all the cases. However, the list of suggested etiologies can be presented. We added S8 Table, “Possible etiology of the confirmed cerebral venous sinus thrombosis with thrombocytopenia cases”.

We thank for this suggestion. In the original manuscript, platelet count nadir ± 14 days of episode start, per cubic mm, median (range) for the grouped episodes are given (**Table 2**). We have now included the suggested data in its footnote: “^f^ For the 98 episodes with Confirmed CVST but without thrombocytopenia these where 239,000 (156,000 – 647,000) and the 1st and 3rd quartile limits where 205,000 and 286,000.

”

*Reviewer #2 -6.*

*A more recent study which was based on pharmacovigilance data from the EEA (doi: 10.1212/WNL.0000000000013148) provides more updated information than the EMA report that has been cited in Supplementary table 1 (reference 6). Consider updating that.*

Response  *#2 -*6: We currently have updated this reference.

*Reviewer #2 -*7.

Previous studies, such as doi: 10.1212/WNL.0000000000013148, have also not found an increased risk of CVT in recipients of mRNA vaccines against SARS-CoV-2. Consider discussing that.

Response #2 -7: In Discussion, Line 269, we added this reference in the sentence: “Notably, confirming the findings of others [32,33]”

*Reviewer #2 -*8.

No data is provided for Ad26.COV2.S. The reasons for this should be explained.

Response #2 -8. Line 61 now states: “Exposures to COVID-19 vaccine brands other than displayed were extremely rare.”
