# Supplementary material for: Incidence of sinus thrombosis with thrombocytopenia—A nation-wide register study
Source: PLoS One. 2023 Feb 24;18(2):e0282226. doi: 10.1371/journal.pone.0282226 (PMC9956025; doi:10.1371/journal.pone.0282226)
Supplement: Attachment — Submitted filename: Response to the Reviewers 2023-01CLEAN.docx [file pone.0282226.s010.docx]

# Response to Reviewers

08 Jan 2023

PONE-D-22-09809R1

Incidence of Sinus Thrombosis with Thrombocytopenia - a Nation-wide Register Study

PLOS ONE

Dear Editor,

Again, we thank your team including the Reviewers for their work on our manuscript and are happy to submit a second revised manuscript. As requested, we respond to each of your comments, and we now include both 'Revised Manuscript with Track Changes and an unmarked version of our revised paper ‘Manuscript’. We include the Editors’ and Reviewers’ comments in *italics* within quotes.

## Responses to comments by Reviewer #1

“*Reviewer #1: Dear Editor,
 I still have some concerns about the manuscript:

Response #1 – 5
 The authors response is ambiguous. They should directly declare in the manuscript why
 they had 167 register-based CVST episodes in 156 subjects in a 15-month period of observation in the first revision.
 The changed manuscript ‘In our data, 2 of the 117 patients presented with more than a single [confirmed CVST] episode during the study period.’ did not reply my argument*”

**Response #1 – 5 R1 08JAN2023.**

In Comment #1-5 on our primary submission, reviewer #1 argued that having 11 recurrencies after CVST all within the same 15-month recruitment period would be unusual. We agree.

The reason is obvious: these 11 episodes were mostly not true recurrencies. We have now included more info in the manuscript right where the numbers 167 episodes in the 156 subjects are first stated. That is, we added some details of the register-based episodes that were not confirmed in the chart review. The seemingly high recurrence rate was true only for the register-based definition and, in fact, was not existent for the 117 episodes fulfilling the requirements of ‘*confirmed* CVST’.

Our new addition (as a tracked change, Results, row 170):

“Altogether, we detected **167** *register-based CVST* episodes in 156 subjects (Fig 1). Of these, 50 episodes in 41 patients were not novel cases of a radiologically confirmed CVST in our patient file review. The remaining 117 (70%) in 115 patients were classified as c*onfirmed CVST*.”

“*Response #1 – 6:
 I agree that ‘We only detected two cases and were unable to perform formal analysis that take concomitant disease into account’. However, the authors should clearly declare in the method part that conditions which can induce both CVST and thrombocytopenia was excluded for diagnosing VITT.*”

**Response #1 – 6 R1 08JAN2023.**

Our definition of *confirmed CVST with thrombocytopenia* included a novel CVST with radiological confirmation and thrombocytopenia. This was done without excluding any cases with conditions that could simultaneously induce these two. As such, it fulfilled the requirements of the current Brighton collaboration TTS definition from Nov 2021, ref 7, available from: <https://brightoncollaboration.us/thrombosis-with-thrombocytopenia-syndrome-interim-case-definition/>.

Although not excluding episodes with any *a priori* list of acute conditions that could induce thrombocytopenia we report them (see below). Please note that within our first resubmission, as a response to reviewer comment #2 -5, we also added more data on the 18 episodes with ‘*confirmed CVST with thrombocytopenia’* (S8 Table).

We now also refer to ‘S8 Table Possible etiology of the confirmed cerebral venous sinus thrombosis with thrombocytopenia cases’ from the text (Results, row 175):

“During 3 of the 18 episodes, acute concomitant disease likely to contribute to the thrombocytopenia was diagnosed (infection, operation and head trauma, S8 Table).”

We understand that in three instances, our wording “Concomitant disease” needs the word “chronic” to distinguish it from acute concomitant conditions and disease:

- Subheading in Methods, row 120
- Table 2, 3^rd^ row
- Table 2, footnote ^a^, row 199
